# Mild liver dysfunction in Klinefelter syndrome is associated with abdominal obesity and elevated lipids but not testosterone treatment

**DOI:** 10.1007/s40618-024-02394-3

**Published:** 2024-05-30

**Authors:** C. M. Øzdemir, L. O. Ridder, S. Chang, J. Fedder, J. Just, C. H. Gravholt, A. Skakkebæk

**Affiliations:** 1 Department of Endocrinology and Internal Medicine and Medical Research Laboratories, Aarhus University Hospital, Palle Juul-Jensens Boulevard 99, 8200 Aarhus N, Denmark; 2 Centre of Andrology and Fertility Clinic, Department D, Odense University Hospital, Odense, Denmark; 3 Research Unit of Human Reproduction, Institute of Clinical Research, University of Southern Denmark, Odense, Denmark; 4 Department of Molecular Medicine, Aarhus University Hospital, Aarhus, Denmark; 5 Department of Clinical Medicine, Aarhus University Hospital, Aarhus, Denmark; 6 Department of Clinical Genetics, Aarhus University Hospital, Aarhus, Denmark

**Keywords:** Klinefelter syndrome, Liver dysfunction, Testosterone replacement therapy, Hypogonadism, Obesity, Cholesterol

## Abstract

**Context:**

Klinefelter syndrome (KS) is associated with hypergonadotropic hypogonadism, which contributes to characteristic phenotypical manifestations including metabolic alterations. Extensive research has demonstrated important associations between androgens and liver function.

**Objectives:**

Investigation of the association between metabolic parameters, sex hormones and liver function in males with KS, both treated (T-KS) and untreated (U-KS) and healthy control males.

**Methods:**

A total of 65 KS males were recruited, of which 32 received testosterone replacement therapy (TRT). Also, 69 healthy controls were recruited. We used alanine aminotransferase (ALAT), alkaline phosphatase and PP (prothrombin-proconvertin time ratio) as the main liver markers. Multivariable regression was performed within the three groups. All statistics were calculated using STATA. Principal component analysis was utilized to demonstrate the interconnected patterns among all measured biomarkers, and to elucidate how the different groups were linked to these patterns.

**Results:**

Higher levels of main liver markers were observed in U-KS compared to controls, with no significant differences between U-KS and T-KS. T-KS had lower abdominal fat, total cholesterol, and LDL cholesterol than U-KS. Using multivariable models, variation in ALAT in U-KS was explained by HOMA2%S; in T-KS by BMI and SHBG; and in controls by hip circumference and estradiol. We found no multivariable models explaining variation in PP in U-KS; in T-KS, PP was explained by BMI and LDL cholesterol, and in controls by total cholesterol. Using principal component analysis U-KS was positively associated to D1 (an obese profile, which also included ALAT) and controls negatively associated with D1 (non-obese profile).

**Conclusion:**

KS males have mild liver dysfunction reflected by a significant increase in the main liver markers and decrease in albumin. The presented data underscore a primary role of metabolic conditions including obesity, insulin resistance and unfavourable lipid profile, in the elevated liver function markers seen in males with KS. Whether TRT can improve liver function in KS warrants further studies. Our findings, highlight that an evaluation of the liver function should be part of the clinical care in males with KS.

## Introduction

Klinefelter syndrome (KS; 47, XXY) is a genetic disorder caused by the presence of an extra X chromosome in males [1, 2]. With a prevalence of 1 in every 660 newborn male it is the most common sex chromosome aneuploidy in males [2–4]. Common phenotypical manifestations include tall stature, small testes, gynecomastia, hypergonadotropic hypogonadism and infertility [5, 6]. Further, KS is associated with an increased morbidity [7, 8], including a significantly increased risk of liver cirrhosis, metabolic syndrome, decreased insulin sensitivity and abdominal adiposity [9], leading to a loss in lifespan of 2–5 years [10–12].

Testosterone replacement therapy (TRT) is a central part in clinical care for males with KS due to hypergonadotropic hypogonadism [6, 13, 14]. TRT increases lean body mass (LBM) and reduces fat mass [15], but also reduces the development of metabolic syndrome and insulin resistance [16, 17]. The physiological effects of testosterone promote cholesterol storage, reduction in glucose uptake and lipogenesis in the liver [18].

A recent population-based cohort study showed that elevated liver parameters was twice as common among children and adolescents with KS compared to controls and was linked to the presence of overweight and obesity [19], indicating liver dysfunction at an early age in KS. However, in a retrospective study of patients with KS in an outpatient setting, liver parameters among KS receiving TRT were within normative ranges [13]. Thus, the prevalence of elevated liver enzymes and liver dysfunction in KSs is largely unknown. In addition, it is not known whether TRT may have a beneficial effect on liver function in males with KS.

Here, we present data on markers of liver function, including liver enzymes and the potential impact of TRT, anthropometry, body composition, lipid metabolism, insulin resistance and sex hormones in 65 patients with KS and 69 age-matched controls.

## Research design and methods

### Participants

A total of 65 KS males were recruited from endocrine and fertility clinics. The inclusion criteria were age above 18 years and verified KS karyotype. Exclusion criteria were untreated hypothyroidism or hyperthyroidism, present or previous malignant disease, clinical liver disease, or treatment with drugs known to interfere with glucose homeostasis or fat metabolism. Data from this study regarding glucose metabolism, insulin resistance, anthropometry and bone mineral density have previously been reported [9, 20].

**Fig. 1:**
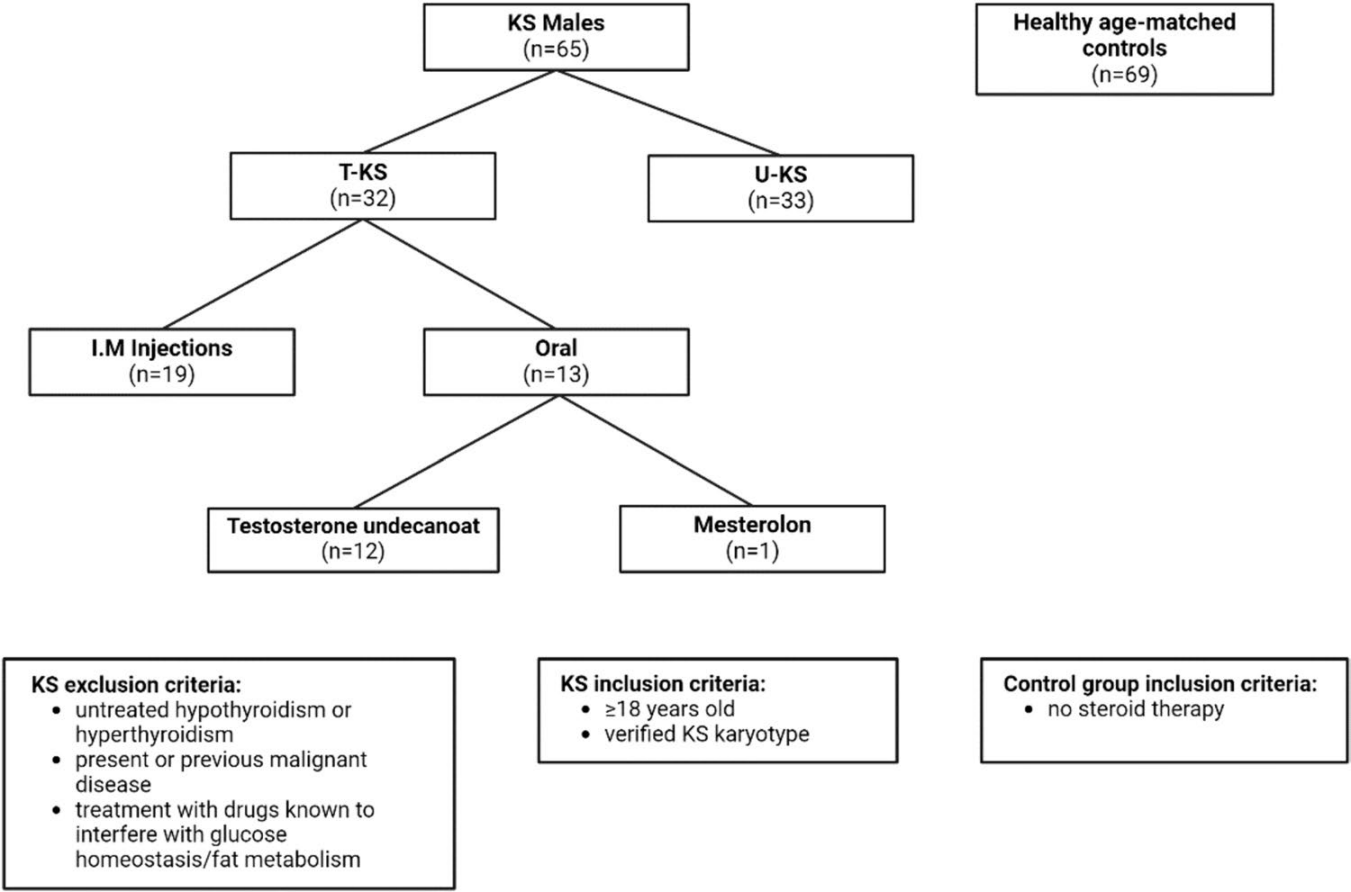
Flow diagram with an overview of the therapy forms, and the inclusion and exclusion criteria for the included KS males and healthy age-matched controls. I.M: intramuscular; T-KS: individuals receiving testosterone replacement therapy; U-KS: individuals not receiving testosterone therapy; KS: Klinefelter syndrome

In this cohort, 32 KS (49.2%) males received TRT (T-KS) at the time of investigation (intramuscular testosterone injections (n = 19), oral testosterone undecanoate (n = 12) and mesterolon (n = 1). We do not have information concerning the exact date for the last injection, due to the inability of some KS patients to recall, and since we did not have access to all patient files (Fig. 1).

Of the 33 KS males without TRT (U-KS), none had received TRT during the last year before examination. The 69 healthy agematched control men were recruited by advertising for healthy volunteers at Aarhus University and at the Blood Bank at Aarhus University Hospital. None of the healthy controls received any kind of steroid therapy.

## Methods

All participants were examined in the morning after an overnight fast.

### Blood samples

Venous blood samples were drawn, and serum and plasma were immediately separated and stored at −20 °C in multiple vials for later analysis.

### Anthropometric measures

Body weight was measured to the nearest 0.1 kg and height was measured to the nearest 0.5 cm. A tape measure was used for hip and waist measurements.

### Whole-body dual-energy x-ray absorptiometry

Whole-body dual-energy x-ray absorptiometry (DXA) scans were performed on a Hologic 2000/w osteodensitometer (Hologic Inc., Waltham, MA, USA), and lean body mass (LBM) (kg), total body fat (%) and abdominal fat (%) were calculated as previously reported [13].

### Assays

We studied liver function markers, here defined as alanine-aminotransferase (ALAT), alkaline phosphatase, bilirubin, and prothrombin-proconvertin time ratio (PP) which were determined on a Cobas Integra (Roche). Testosterone, oestradiol, albumin, sex hormone binding globulin (SHBG), cholesterols, plasma glucose, insulin and the homeostasis model assessment (HOMA = $\frac{Insulin Glucose}{22.5}$) insulin sensitivity index HOMA2%S (calculated using calculator from https://www.rdm.ox.ac.uk/about/our-clinical-facilities-and-units/DTU/software/homa) [9, 21], where also measured. Testosterone was estimated by a method described by Bartsch [22].

## Statistics

The statistical analyses were performed in two steps. First, comparisons were made between U-KS and T-KS. Next, comparisons were made between U-KS and controls. This was done to study the possible effects of testosterone within the KS group and to study the effect of having KS without receiving TRT.

Continuous variables were tested for normality using the Kolmogorov–Smirnov test. Differences between groups were analysed with a parametric Student’s t-test for normally distributed data, and with a Mann–Whitney-Wilcoxon test for non-normally distributed data.

ALAT, alkaline phosphate and PP were the main outcome parameters and were used as dependent variables in the ensuing analysis.

To evaluate the impact of the independent variables on the dependent variables we used stepwise multivariable regression analysis on each of the dependent variables individually, this method allows one to check for multicollinearity. First, we performed multivariable analysis within the three groups of participants (U-KS, T-KS and controls), at first data were normalized, however, as this did not seem to impact analysis, we kept the original scales for readability purposes. To select principal independent variables for the multiple regression analyses, we used Spearman correlation analysis. From these correlation analyses, we used the most significantly correlated variable and the most clinically relevant, from each of the five subcategories of variables—body composition, lipid metabolism, insulin resistance, sex hormones, and anthropometric (Table 1). Therefore, a maximum of five variables entered the multiple regression model. Significance level for entering and for removal of variables from the model was P < 0.05 and P < 0.10, respectively. P-values lower than 0.05 were considered significant. All statistics were calculated using STATA 17.0 (StataCorp LLC, College Station, TX, USA).

**Table 1:** Anthropometry and biochemistry in U-KS and T-KS males and controls

	U-KS	T-KS	Controls	P-value	
	n = 33	n = 32	n = 69	U-KS vs. T-KS	U-KS vs. controls
*Anthropometry*					
Age (years)	34.8 (19.0–66.2)	36.4 (19.3–62.3)	36.4 (19.2–68.0)	0.81	0.82
Height (cm)	184.0 ± 9.0	185.1 ± 8.1	181.1 ± 5.7	0.62	0.1
Weight (kg)	92.5 (65.8–168.9)	84.7 (56.8–149.8)	82.4 (60.1–112.0)	0.61	< 0.001
BMI (kg/m^2^)	27.1 (20.0–60.6)	24.9 (18.1–54.7)	24.9 (19.0–31.6)	0.45	0.01
Waist circumference (cm)	109.0 (81.0–179.0)	103.3 (80.5–175.0)	91.8 (76.0–122.0)	0.34	< 0.001
Hip circumference (cm)	103.0 (83.0–166.0)	101.0 (83.5–145.0)	90.5 (80.0–114.0)	0.49	< 0.001
*Body composition*					
Total body fat (%)	28.5 ± 7.7	23.8 ± 10.2	18.6 ± 6.7	< 0.05	< 0.001
Abdominal fat (%)	31.9 (10.1–49.3)	22.6 (2.4–56.2)	17.1 (3.2–43.7)	0.07	< 0.001
Lean body mass (kg)	68.5 ± 7.3	73.2 ± 9.7	78.7 ± 6.3	< 0.05	< 0.001
*Lipid metabolism*					
Triglycerides (mmol/l)	1.5 (0.4–40.2)	1.6 (0.6–4.5)	0.8 (0.4–2.5)	0.79	< 0.001
Total cholesterol (mmol/l)	5.9 (3.7–13.5)	5.1 (3.8–7.4)	4.7 (3.3–7-4)	0.03	< 0.001
HDL cholesterol (mmol/l)	1.0 (0.5–2.2)	0.9 (0.6–1.9)	1.3 (0.8–2.6)	0.25	< 0.001
LDL cholesterol (mmol/l)	3.7 ± 0.8	3.2 ± 0.7	3.2 ± 0.9	0.01	0.003
*Liver function*					
Alanine aminotransferase (U/l)	28 (13–147)	34 (12–72)	21 (12–124)	0.98	< 0.01
Alkaline phosphatase (U/l)	146 (50–253)	138 (55–309)	109 (43–267)	0.93	0.02
Bilirubin (µmol/l)	15 (6–31)	16 (8–35)	16 (5–63)	0.27	0.35
PP	1.25 (0.20–1.30)	1.19 (0.65–1.30)	1.04 (0.46–1.30)	0.25	< 0.001
Albumin (µmol/l)	667.8 ± 48.5	685.3 ± 45.0	698.0 ± 54.0	0.14	< 0.01
*Sex hormones*					
Testosterone (nmol/l)	12.9 (0.8–37.3)	14.3 (1.9–72.2)	22.8 (12.0–55.5)	0.22	< 0.001
Free testosterone (nmol/l)	0.3 (0.0–0.7)	0.3 (0.1–2.6)	0.5 (0.2–1.4)	0.17	< 0.001
Oestradiol (pmol/l)	77 (40–140)	89 (44–290)	81 (40–210)	0.06	0.73
SHBG (nmol/l)	31.0 (16.0–79.0)	30.5 (13.0–87.0)	36.0 (22.0–99.0)	0.60	0.02
*Additional Biochemistry*					
Homa2%S (%)	67.3 (17.7–301.0)	88.8 (11.7–358.6)	129.7 (34.4–513.7)	0.64	< 0.001

An overview of the anthropometry and biochemistry in U-KS and T-KS males and controls. Data are medians (total range) or means ± SD. *BMI* body mass index; *HOMA2%S* HOMA of insulin sensitivity; *PP* prothrombin-proconvertin time ratio; *SHBG* sex hormone-binding globulin

Principal component analysis.

To elucidate linear correlations between the various clinical measurements, we employed unsupervised clustering using principal component analysis. Principal component analysis condenses a set of standardized variables into a smaller number of components, or dimensions, called principal components. These components are combinations of the original variables and capture the underlying data variance, revealing patterns that might not be apparent in the individual measurements. As such, these components capture the essential information in the data, allowing identification of trends and relationships. In addition, this method enables us to explore and visualize how the different groups are associated with these patterns. To implement principal component analysis, we used the R package FactoMineR [23]. Missing values were handled by imputing them with the respective variable means.

## Results

The liver function markers were significantly higher in U-KS (all ps < 0.05) while albumin was lower in U-KS (p < 0.01), when compared with controls (Table 1). As previously reported, total body fat, abdominal fat, the anthropometric and lipid profile variables were higher among U-KS (all ps < 0.05), whereas LBM, HDL cholesterol, HOMA2%S and sex hormones were lower in U-KS (all ps < 0.05), when compared to controls (Table 1).

There was no difference in the liver function markers, anthropometric variables, sex hormones and HOMA2%S between U-KS and T-KS. Total body fat, total cholesterol and LDL cholesterol were significant lower (ps < 0.05), and LBM higher (p < 0.05) among T-KS compared to U-KS (Table 1).

Significant correlations were observed for the liver function markers and the 16 variables within each of the three groups U-KS, T-KS and controls using Spearmen analysis (p < 0.05 and p < 0.01) (Table 2). In U-KS, ALAT was correlated with abdominal fat, HOMA2%S and SHBG. Alkaline phosphatase was correlated with bilirubin, HDL cholesterol, LDL cholesterol and oestradiol. Lastly, PP was correlated with BMI, abdominal fat, triglycerides, total cholesterol and HOMA2%S.

**Table 2:** Data concerning anthropometry, body composition, lipid metabolism and sex hormones, which previously have been reported^9^ and are reported here as supportive data

Dependent variable ►	Alanine aminotransferase			Alkaline phosphatase			PP		
Independent variables ▼	U-KS N = 33	T-KS N = 32	Control N = 69	U-KS N = 33	T-KS N = 32	Controls N = 69	U-KS N = 33	T-KS N = 32	Controls N = 69
Alanine aminotranferase	1								
Alkaline phosphatase	0.029	**−** 0.04	0.27^*^	1					
Bilirubin	0.01	−0.36^*^	**−** 0.01	0.51^**^	0.39^*^	0.45^**^			
PP	0.26	0.55^**^	0.15	**−** 0.15	**−** 0.12	**−** 0.23	1		
Weight	0.09	0.57^**^	0.34^**^	**−** 0.18	**−** 0.20	**−** 0.04	0.14	0.51^**^	0.25^*^
Waist circumference	0.24	0.50^**^	0.34^**^	0.33	**−** 0.09	0.11	0.19	0.57^**^	0.36^**^
Hip circumference	0.17	0.37^*^	0.37^*^	0.28	**−** 0.02	0.23	0.06	0.42^*^	0.24
BMI	0.13	0.60^**^	0.34^**^	– 0.05	**−** 0.11	**−** 0.02	0.40^*^	0.61^**^	0.38^**^
Total body fat	0.21	0.48^*^	0.28^*^	0.02	**−** 0.13	**−** 0.03	0.29	0.51^**^	0.37^**^
Abdominal fat	0.38^*^	0.50^*^	0.31^*^	0.04	**−** 0.07	**−** 0.04	0.41^*^	0.55^**^	0.41^**^
Lean body mass	**−** 0.22	-0.46^*^	−0.27^*^	**− **0.03	0.13	0.03	**−** 0.27	−0.50^**^	−0.37^**^
Triglycerides	0.23	0.45^*^	0.17	**−** 0.11	**−** 0.18	**−** 0.10	0.54^**^	0.47^**^	0.22
Total cholesterol	0.15	0.18	0.19	**− **0.30	**−** 0.22	**−** 0.11	0.35^*^	0.51^**^	0.34^**^
HDL cholesterol	**−** 0.29	−0.36^*^	**−** 0.20	0.41^*^	0.12	**−** 0.14	**−** 0.25	−0.40^*^	**−** 0.08
LDL cholesterol	0.18	0.22	0.21	−0.40^*^	**−** 0.31	**−** 0.06	0.16	0.54^**^	0.33^**^
HOMA2%S	−0.61^**^	−0.66^**^	**−** 0.20	**−** 0.10	**−** 0.02	−0.26^*^	−0.41^*^	−0.3^*^	**−** 0.23
Albumin	0.03	0.17	0.10	**−** 0.00	**−** 0.11	0.50^**^	**−** 0.30	**−** 0.01	−0.29^*^
Testosterone	0.03	**−** 0.09	0.09	0.14	**−** 0.17	0.07	**−** 0.32	**−** 0.05	**−** 0.03
Free testosterone	0.27	0.04	**−** 0.04	0.01	**−** 0.14	0.03	**−** 0.21	0.01	**−** 0.10
SHBG	−0.35^*^	−0.45^*^	0.07	0.05	**−** 0.14	**−** 0.03	**−** 0.29	-0.31	0.04
Oestradiol	0.05	0.27	0.27^*^	0.48^**^	0.04	0.05	**−** 0.13	0.35^*^	**−** 0.20

Using spearmen correlation analysis, on all patients and controls included. ^*^p < 0.05; ^**^p < 0.01 Table showing the correlation between alanine aminotransferase, alkaline phosphatase and PP and anthropometry, body composition, lipid metabolism and sex hormones. *BMI* body mass index; *HOMA2%S* HOMA of insulin sensitivity; *PP* prothrombin-proconvertin time ratio; *SHBG* sex hormone-binding globulin

In T-KS, ALAT correlated with bilirubin, PP, weight, waist circumference, hip circumference, BMI, body composition variables, triglycerides, HDL cholesterols, HOMA2%S and SHBG. Alkaline phosphatase correlated only with bilirubin (Table 2). PP correlated with the anthropometric variables, body composition and lipid profile variables, HOMA2%S and oestradiol (Table 2). In controls, ALAT correlated with alkaline phosphatase, oestradiol, anthropometric and body composition variables. Alkaline phosphatase correlated with bilirubin, HOMA2%S and albumin, and PP correlated with weight, waist circumference, BMI, body composition variables, total cholesterol, LDL cholesterol and albumin.

Testosterone and free testosterone did not correlate with any of the liver function markers (Table 2).

## Multivariable regression models to predict independent variables of the liver function markers

In U-KS ALAT was predicted by HOMA2%S. In T-KS SHBG and BMI were significant contributors to the model and accounted for 43% of the variability in ALAT. For the control group hip circumference and oestradiol were significant contributors and accounted for 18% of the variability in ALAT. No multivariable models explained the variance in PP in U-KS. In T-KS, BMI and LDL cholesterol were significant contributors and accounted for 34% of the variability in PP. For controls PP was predicted by total cholesterol.

No multivariable models explained the variance in alkaline phosphatase in U-KS, T-KS or controls. (Table 3).

**Table 3:** Multiple regression models for ALAT and PP in U-KS, T-KS males and controls

For ALAT as dependent variable			
Independent variables	Coefficient	95%CI	P value
U-KS n = 31		adjusted R^2^ = 19%	
HOMA2%	−0.24	−0.4;−0.07	0.008
T-KS n = 31		adjusted R^2^ = 43%	
BMI	1.1	0.5;1.6	< 0.001
SHBG	−0.3	−0.6;−0.1	0.01
Controls n = 66		adjusted R^2^ = 18%	
Hip circumference	0.95	0.42;1.49	0.001
Oestradiol	0.12	−0.003;0.24	0.06

For PP (prothrombin-proconvertin time ratio) as dependent variable				
Independent variables	Coefficient	95%CI		P-value
T-KS n = 30		adjusted R^2^ = 0.34		
BMI	0.008	0.001;0.015		0.027
LDL cholesterol	0.095	0.02;0.17		0.017
Control males n = 67		adjusted R^2^ = 12%		
Total cholesterol	0.066	0.02;0.11		0.003

Multiple regression models for ALAT and PP in treated and untreated KS males and controls. *BMI* body mass index; *HOMA2%S* HOMA of insulin sensitivity HOMA2%: *SHBG* sex hormone-binding globulin

## Principal component analysis for visualizing dimensions influencing liver function markers

This study contained a large dataset (high-dimensional) with a high number of clinical variables. To visualize and interpret our high-dimensional data in a more manageable, lower-dimensional format, principal component analysis was conducted using all the variables as input.

**Fig. 2:**
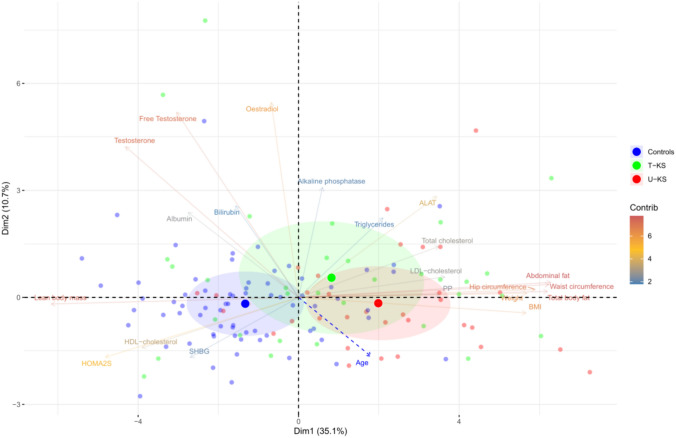
Principal component analysis of variables contributing to variation in liver parameters. Principal component analysis of clinical variables contributing to variation in liver function markers. Red dots are untreated KS males (U-KS), green dots are treated KS males (T-KS), and blue dots are controls. ALAT: alanine aminotransferase; BMI: body mass index; D1: Dimension 1; D2: Dimension 2; HOMA2%S: HOMA of insulin sensitivity; PP: prothrombin-proconvertin time ratio; SHBG: sex hormone-binding globulin

Principal component analysis identifies the most influential components, consisting of a combination of variables, that account for the variability in the data, simplifying its complexity while preserving essential patterns and relationships. Dimension 1 (D1) explained 35.1% of the variance, while dimension 2 (D2) explained 10.7% (Fig. 2**).** It was evident that the anthropometric and body composition variables best described D1. The anthropometric and body composition variables all had a strong positive correlation with this dimension, while LBM was negatively correlated to this dimension (Table 4**)**. This supported our findings above, as the U-KS group was positively associated to D1 (obese profile, p value 5.01 $\times$10^–7^), whereas controls were negatively associated to D1 (non-obese profile, p-value 5.02 $\times$10^–10^) (Table 4). The T-KS group clustered in-between the U-KS and controls, suggesting an anthropometric and body composition normalization. Besides the anthropometric and body composition variables, ALAT, total cholesterol and PP were also positively associated with D1, and as such, with increased obesity. On the other hand, sex hormones, HDL cholesterol and HOMA2%S were negatively correlated with D1, and thus, with decreased obesity. None of the groups were significantly associated with D2.

**Table 4:** Correlations and dimensions

	Correlation to Dimension 1	P value
Abdominal fat	0.861307	1.23E-40
Waist circumference	0.854316	2.47E-39
Total body fat	0.849859	1.55E-38
Hip cicumference	0.808304	3.66E-32
Weight	0.779736	1.27E-28
BMI	0.776262	3.16E-28
LDL cholesterol	0.499495	8.06E-10
PP	0.480471	4.22E-09
Total cholesterol	0.478191	5.12E-09
ALAT	0.471745	8.73E-09
Triglycerides	0.288321	7.29E-04
Age	0.247094	4.00E-03
Alkaline phosphatase	0.0815	3.49E-01
Oestradiol	−0.09342	2.83E-01
Bilirubin	−0.21494	1.26E-02
SHBG	−0.37	1.08E-05
Albumin	−0.37718	7.05E-06
Free testosterone	−0.41642	5.60E-07
HDL cholesterol	−0.53498	2.76E-11
Testosterone	−0.59103	5.57E-14
HOMA2%S	−0.6596	4.45E-18
Lean body mass	−0.84487	1.13E-37
U-KS	1.493605	5.01E-07
T- KS	0.328309	5.06E-02
Controls	−1.82191	5.02E-10

Principal component analysis. An overview of correlation to dimension 1. *ALAT* Alanine aminotransferase; *BMI* body mass index; *HOMA2%S* HOMA of insulin sensitivity; *PP* prothrombin-proconvertin time ratio; *SHBG* sex hormone-binding globulin

## Discussion

The present study showed that liver function markers are significantly higher in males with KS compared to healthy age-matched control males indicating mild liver dysfunction in KS. In the literature, only a few studies have included data on liver function in KS, thus our knowledge in this field is sparse. Davis et al. [19] showed in a recently published study, that elevated liver parameters was twice as common among children and adolescents with KS suggesting that liver dysfunction is set early on in life. Further evidence of liver dysfunction in KS come from a nationwide cohort study of 832 males with KS reporting a 2–threefold higher risk of cirrhosis of the liver in KS males [7].

Our comparison between T-KS and U-KS revealed no significant difference in liver function markers. This is in contrast to the results of a retrospective study of patients with KS in an outpatient setting [13]. Here liver parameters among KS receiving TRT were within normative ranges. Studies in hypogonadal men have also demonstrated an improvement in liver function after two years of oral testosterone or when increasing the level of testosterone [24, 25]. However, the present data did show a trend towards improvement in liver function in T-KS males indicating that TRT may improve liver function in males with KS. The lack of significance may be due to our small sample size.

To investigate associations that could give insight into the cause of mild liver dysfunction in KS and identify risk factors, we performed multivariable and principal component analysis. Here, mild liver dysfunction was associated with increased anthropometric variables, total body fat, abdominal fat, triglycerides, total cholesterol, and increased insulin resistance, underscoring a primary role of metabolic conditions in elevated liver function markers in males with KS. This is consistent with the findings by Davis et al. who demonstrated a link to the presence of overweight and obesity [19]. The associations and risk factors of mild elevated liver function markers identified in this study are not specific for KS, as these have also been described in the general population [26]. However, the mean age in our cohort is between 34.8 and 36.4 years and as such the elevated liver function markers occur earlier than seen in the general population in agreement with the results by Davis et al. [19].

The multivariable analysis identified BMI and SHBG as the strongest predictor for increased ALAT in T-KS. Obesity is also a major risk factor in developing non-alcoholic fatty liver disease where increased ALAT levels are known to be a predictor of the disease [27].

BMI is known to be associated with metabolic syndrome and increased ALAT levels [28], also showing a positive correlation in our model. On the other hand, SHBG displayed a negative correlation with ALAT. Previous research has also reported that reduced SHBG levels increase the risk of hepatic steatosis or elevated ALAT levels [29]. This also correlates to the relation between hyperinsulinemia/metabolic syndrome and decreased levels of SHBG [9, 29] seen in KS males.

In U-KS males, HOMA2%S emerged as the only predictor for ALAT showing that increased insulin resistance was associated with increased ALAT. In the control group, higher levels of ALAT were associated with hip circumference and oestradiol levels accounting for 17% of the variation in ALAT. Increased hip circumference is commonly linked to the metabolic syndrome, as well as increased oestrogen levels from adiposity which in turn can lead to hepatic dysfunction and increased ALAT levels [30, 31].

Alkaline phosphatase was associated with several clinical measurements, however prediction models with the current set of variables was not possible. Increased alkaline phosphatase was observed in U-KS compared to controls, likely derived primarily from bone and not the liver. We have previously shown that bone-specific alkaline phosphatase is slightly higher in KS and predicts some aspects of bone metabolism [20]. Decrased bone mineral density is frequently seen in patients with KS and may be attributed to hypogonadism [32], but also the increased fat body mass as fat tissue release proinflammatory mediators can stimulate osteoclast activity leading to bone resorption [33].

We found that PP are elevated in KS individuals. It is worth noting that more KS males reached the upper measurement limit (upper limit 1.3) compared to controls, which could potentially underestimate the observed difference. We did not find that TRT normalized PP levels. However, we did find a negative correlation between HDL cholesterol and PP levels in KS males, but not in the control group. We did find that total cholesterol, LDL cholesterol and HDL cholesterol was associated with PP values, in particular HDL cholesterol was negative associated with PP values in KS males in particular T-KS males. This increased level of PP should, in this study, mainly be considered as a marker of liver synthesis. KS males are associated with an increased risk of thromboembolism, that could partially be explained by decreased fibrinolytic capacity. The presence of a hypercoagulable state Is still debateable; however, studies indicate that untreated KS could be affected by reduced activation of the protein C anticoagulant pathway, reflected by increased Calibrated automated thrombography (CAT) assay thrombin generation in the presence of thrombomodulin [12, 15, 34].

Surprisingly, HDL cholesterol was correlated with several predictors for impaired health in KS males, but not in controls suggesting an HDL cholesterol could be a possible overall biomarker for treatment of KS males. A recent study has showed that individuals with chronic liver disease have decreased levels of HDL cholesterol which can contribute to the severity of the disease. This is an interesting predictor for disease progression [35].

It is important to note the limitations of this study. The participants did not undergo liver biopsies or ultrasonography to identify early non-alcoholic fatty liver disease changes in liver which could give biased biochemistry results as they are considered healthy. For instance, a recent RCT-study found no improvements in liver measurements after receiving testosterone treatment or placebo [36]. Future studies should include ultrasonography of the liver to investigate if early changes of non-alcoholic fatty liver disease are seen in males with KS. In addition, longitudinal studies could contribute to our understanding of the natural history of the liver dysfunction in males with KS and associated liver pathologies. The limited size of the overall study group is a weakness and future studies should include larger study groups.

In conclusion, males with KS have a mild liver dysfunction, reflected by a significant increase in ALAT, alkaline phosphatase, PP and decreased levels of albumin, compared to an age matched control group. The presented data underscore a primary role of metabolic conditions including obesity, insulin resistance and unfavourable lipid profile, in the elevated liver function markers seen in males with KS. Whether TRT can improve liver function in KS warrants further studies. Our finding, highlight that an evaluation of the liver function should be part of the clinical care in males with KS.

## Data Availability

The datasets generated during and/or analysed during the current study are available from the corresponding author on reasonable request.

## Abbreviations

ALAT: Alanine aminotransferase
BMI: Body mass index
CVD: Cardiovascular disease
D1: Dimension 1
D2: Dimension 2
DXA: Dual-energy X-ray absorptiometry
HDL: High-density lipoproteins
HOMA: Homeostasis model assessment
HOMA2%S: HOMA of insulin sensitivity
KS: Klinefelter syndrome
LBM: Lean body mass
LDL: Low-density lipoprotein
PP: Prothrombin-proconvertin time ratio
SHBG: Sex hormone-binding globulin
TRT: Testosterone replacement therapy
T-KS: Treated KS patients
TBF: Total body fat
U-KS: Untreated KS patients
